# Factors affecting the protection of data rights in sports events: a configurational analysis

**DOI:** 10.1038/s41598-024-56074-6

**Published:** 2024-03-04

**Authors:** Xiaoyu Li, Xinyan Guo

**Affiliations:** School of Economics and Management, Chengdu Sport University, Chengdu, 610041 Sichuan China

**Keywords:** Environmental sciences, Environmental social sciences

## Abstract

The development of algorithms and the spread of digital infrastructure have contributed significantly to the productivity of the digital economy. Data has come to be known as the “oil of the digital economy”. At the same time, data has begun to participate more deeply in the production activities of the global sports industry chain, and the international discussion on how to protect the rights of sports event data has been increasing. Based on the configurational theory and fuzzy-set qualitative comparative analysis, this study discusses the factors affecting the protection of sports event data rights. The study found three configuration paths for achieving high enterprise data protection effectiveness and two for achieving low enterprise data protection effectiveness. The results of this study provide theoretical support for governments to address the issue of sports event data rights. They will also facilitate the safe use of data in sports, promote the global sports industry and humanitarian action development, and contribute to international sustainable development.

## Introduction

Accompanied by the acceleration of the digitalization process, people's mastery and use of data have made a qualitative leap, and data has gradually become a key production factor in the era of the digital economy. Among them, the commercial value of sports event data has been fully revealed^1^. Even a new industry chain has been extended around sports event data, i.e., sports event "right holders" sell live game data to data companies, distributing the data to end-users, such as the sports betting industry, forming a trillion-dollar sports data industry^2^. However, due to the powerful ability of big data technology to collect, store, and process data, ethical issues such as infringement of athletes' privacy rights and jeopardizing the security of sports data, as well as market issues such as illegal theft of sports data, infringement of intellectual property rights of sports events, and unfair competition, have begun to emerge frequently in sports events^3,4^. In 1997, the case of NBA v. Motorola was brought to the court. The defendant Motorola, without authorization, collected NBA game data from the broadcasting of events and sent it to users' portable communication devices. The case ultimately held that since the game data was in the public domain, it was no longer legally protected. In 2021, a similar situation arose again in China when Shanghai Beitai Electronic Information Technology Co., Ltd (Best Data), as the official data service provider of the CBA, sued Shanghai Xuanti Co. for copying CBA league data by using network crawler technology and providing real-time CBA data to consumers through its official website and mobile phone client "Leisu Sports". In the end, the court held that the defendant's "copying and reproducing" the data collected by the plaintiff damaged the plaintiff's rights and the public interest and constituted unfair competition. The above two cases were decided differently after many years. Still, we can see the necessity of protecting the rights of the sports event data, as well as updating and changing judicial practice.

By sorting out the existing research, the study found that current research mainly focused on five aspects.

1. Data security in sports development. With the arrival of the "big data era", coupled with the proliferation of tracking technology and the desire to monitor human activities, the number, type, and speed of data circulation have increased in geometric progression, and the data security problems in professional sports and sports industry have gradually come to the fore^5^. Statistics and statistically generated information in professional sports are accelerating privatization and commoditization, exacerbating the inequality between "data-rich" elite sports and "data-poor" relatively poor sports. At the same time, with the value-added data resources, the use of data is increasing^6^. At the same time, as the value of data resources increases, the utilization and circulation of data become more frequent, and data security becomes the focus of research^7^. In the sports industry, as it includes the data of the public participating in sports and watching, listening to, and following sports competitions through various media, the security of all these data needs to be improved^8^. In addition, the difficulty in assessing the value of data and the uncertainty of data assets increase the difficulty in determining the cost and contribution of data, which hinders the protection of the rights and interests of all interested parties^9^.
2. Privacy and security of athletes' data^10^. Big data monitoring can capture athletes' biological and real-time data and store the data in the cloud to form an athlete dataset. Algorithms can analyze a series of data to assess the performance of athletes, such as reflecting the player's sports data (passing routes, frequency of passes, etc.), physical functioning, etc., and adding targeted improvements. However, while big data monitoring can help humans make the best judgment, there are also potential disadvantages, e.g., the behavior of some potential customers visiting during a match is doing the business of a data producer^11^. Users unknowingly accept the constant dynamic collection of private data on their habits and behaviors by wearable devices^12^.
3. Data misuse in sports event coverage. Data visualization is a booming area in sports journalism. Sports events often generate a large amount of statistical data. Through the analysis and processing of these data, to distill the content of interest to the audience and dig out the intrinsic correlation behind the data, in-depth analysis of the game of a new type of sports news reporting methods dramatically improves the dissemination of the effect and scope of the game, for example, based on the shooting data of the NBA game made into a shooting area heat map, as a way to analyze the mid-range shooting in NBA games. For example, a heat map of the shooting area based on NBA shooting data is used to analyze the trend of mid-range shooting and three-point shooting in the NBA. However, certain security risks are associated with data use in sports reporting, and the risk of data misuse increases as more data is reused^13^.
4. Data competition in sports betting. Data competition in sports betting occurs primarily between professional leagues and sportsbooks. With the effective legalization of sports betting and the emergence of a premium price for sports data, professional sports leagues claim a property interest in sporting events and the data they generate, and sports betting companies should pay for their officially licensed data. After all, sports betting companies depend on the vast amount of data collected by their leagues and sports data partners for their growth, and charging a fee will help protect proprietary data. Sportsbooks are naturally reluctant to take a cut of the profits. As a result, the practice of selling data collected by leagues or data partners to betting companies at a low price through tracking tools or web-scraping software has emerged. Feld (2020) suggests that federal legislation should address data rights to effectively balance the interests of the leagues and the sportsbooks for this type of problem^2^.
5. Data rights of sports event organizers. The issue of data rights of sports event organizers stems from the ambiguity of the data rights of sports event organizers, which has led to a debate on the allocation of data resources among various stakeholders. Van Rompuy (2014) points out that sports events involve a wide variety of stakeholders, each of which can claim rights or specific interests in the value chain of the organization and utilization of the sports event, for example, clubs, leagues, athletes, federations, fans, media content providers, sponsors, sports facility owners, sports betting operators and the press^14^. In the case of the licensing scheme of the Bundesliga, Germany's elite football league, in this area, for example, tournament owners, when attempting to monetize their commercial interests in sports data, are often faced with conflicts of interest with neighboring industries, such as game publishers, that collect and utilize such data for their economic purposes^15^.

From the point of view of existing studies, there are still many gaps in the research on protecting the rights in sports event data. Existing studies mainly focus on theoretical analysis or factual, empirical studies and propose legislative or judicial response strategies. However, protecting sports event data rights is a systematic project that needs to be focused on at the organizational management level. How to realize the protection of sports event data rights through the role of multiple influencing factors becomes the core issue in this study. The configurational theory and QCA approach provide ideas to address this issue. The configurational theory and QCA method are often used to analyze the causal complexity problem with multiple concurrent factors and are widely used in management research^16^.

In the protection of sports event data rights, athletes, event organizers, data service enterprises (including event service operators, data collection enterprises, data analysis enterprises, etc.), third-party competitors, government departments, and other multi-interested parties must choose to carry out a joint protection strategy to achieve the common goal, i.e., to safeguard their rights and interests. For example, Li (2023) classifies sports data stakeholders into government, enterprises, social public, social organizations, etc., based on the stakeholder strategy management framework proposed by Freeman^4^. However, more than a single factor is needed to determine the prerequisites for realizing such a strategy. For example, the security of user data is affected by the willingness of users and platforms to invest, the cost of investing, the revenue gained, and government incentives^17^. Since protecting data rights in sports events depends on matching multiple factors, the configurational perspective and the QCA approach can provide an adequate explanation. However, for the coupling theory and ecological theory, which also study multi-factors, the advantage of the configurational theory is its concurrent causality, equivalence, and asymmetry. Compared with the coupling and ecological theories, the configurational perspective based on the complexity of causality can distinguish different causal relationships, such as necessary and sufficient, more clearly. This will help to provide a more detailed and in-depth understanding of the causal mechanism of sports event data rights protection^18^.

## Theoretical background and research hypothesis

### Theoretical background

The TOE (Technology-Organization-Environment) framework was proposed by Tornatizky and Fleischer (1990), which emphasizes the division of conditions affecting the application of technology into three categories, i.e., technological, organizational, and environmental conditions. Among them, technical conditions refer to the characteristics of the technology itself, i.e., its relationship with the organization, which focuses on whether the technology matches the organization's structural characteristics and application capabilities and whether it can bring potential benefits to the organization, including technological management capabilities, technological infrastructure, etc.^19^; organizational conditions mainly include organizational size, the scope of operation, institutional arrangements, communication mechanisms, and many other aspects^20^; environmental conditions mainly include the market structure in which the organization is located, the regulatory policies of the external government and other elements^21^.

## Research hypothesis

Referring to existing studies, combining the TOE analysis framework and the practice scenario of sports event data rights protection in China, this study constructs the debugged and expanded TOE framework from the configurational perspective. On this basis, the research hypothesis is proposed. Under the configurational perspective, the conditions do not exist independently but work synergistically through the linkage matching of reinforcement or offset. Therefore, the study will use correlation analysis and regression analysis to verify whether the hypothesis is valid.

First, technical conditions. This includes two secondary conditions: the construction of enterprise technology infrastructure and enterprise technology management capacity. The construction of enterprise technology infrastructure and enterprise technology management capacity mainly refers to the information technology capacity of the enterprise authorized by the event organizer to provide data services. Core information technology is an important driving force in cultivating an enterprise's core competence and can be an essential part of that competence^22^. For example, the number of AI enterprises with deep learning technology as their core competence entered a rapid growth stage after 2010^23^. Sports are no exception, such as Sportradar, a sports event data enterprise built using crawler technology, which provides services to detect abnormal odds and betting volume changes in sports betting^24^. Such enterprises in China include BetaTech, SportsDT, Flying Whale, etc., which provide service content such as event data collection, analysis, and reporting based on their algorithmic models for event organizers, broadcasters, competition departments, teams, and spectators. Therefore, the construction of enterprise technology infrastructure mainly refers to the technical level of event data service enterprises in data collection, processing, application, and protection.

Enhancing the ability of enterprise technology infrastructure construction can improve the data service level of enterprises and then enhance the ability to protect the rights of sports event data and move the infringement risk gate forward^25^. However, the advantage of technology infrastructure is broader than the technology itself. Still, it depends on whether the enterprise can use the existing resources or capabilities to maintain its competitive advantage, which also requires developing and applying the enterprise technology management capability^26,27^. Enterprise technology management ability mainly refers to the management ability of the event data service enterprise to integrate various information and resources, rapidly respond to external changes, and serve the strategic decision-making of the enterprise^28^. In protecting sports event data rights, data service enterprises maximize their technical facility advantages by improving their technical management capabilities to use and protect data effectively. Realizing sports event data rights protection requires the technical support of data service enterprises and the support of the event organizer's technical infrastructure to form adequate security at the data source. The technical infrastructure of event organizers mainly refers to the technical level of event organizers in data collection, processing, application, and protection. Due to the lack of professional and technical personnel and the constraints of multiple event organizations, event organizers often seek external technical support, such as through "service outsourcing" and other forms of cooperation with data service enterprises^28^. However, according to the development of sports events in China, event organizers mostly rely on their information technology support during athletes' essential information collection phase. In this process, the technical infrastructure capacity of event organizers will impact the strength of athletes' privacy protection.

- H1: The construction of enterprise technology infrastructure has a positive effect on enterprise data protection effectiveness.
- H2: The enterprise technology management capacity has a positive effect on enterprise data protection effectiveness.

Second, organizational conditions. Specifically, it includes two secondary conditions: the enterprise allocation of attention to data protection and the enterprise data management system. Organizational conditions usually have corporate scale, business scope, institutional arrangement, communication mechanism, etc. Researchers also often consider the impact of enterprise organizational factors on enterprise technological innovation from the organizational and management level^29,30^. However, in protecting data rights in sports events, in addition to technical conditions, data protection awareness is a more critical factor^31^. The "attention" of data service enterprises to data protection will directly determine enterprises' priority to a series of matters and resources and form solid external protection^29^. In addition, the data management system mainly emphasizes defining and regulating data quality, data standards, data update rate, and the degree of openness of the data itself^25^. In protecting sports event data rights, data service enterprises and event organizers need to clearly define the scope of data management and coverage to improve the encryption and security of event information integration and dissemination^32^. Among them, the enterprise data management system emphasizes data monitoring and management within the organization, such as formulating monitoring strategies in the security area, network resources, hosts, and virtual environments to prevent third parties from infringing^31^.

- H3: The enterprise allocation of attention to data protection has a positive effect on enterprise data protection effectiveness.
- H4: The enterprise data management system has a positive effect on enterprise data protection effectiveness.

Third, environmental conditions. Specifically, it includes three secondary conditions: tournament organizer data management system, government regulation, and government support. Compared with the enterprise data management system, the tournament organizer data management system emphasizes controlling data sharing scope and authority, such as determining the scope and content of event data cession. As the influence of sports events increases, the sports event data industry has become a multi-billion-dollar industry, and the vast potential of China's sports event data industry has begun to stand out. In 2018, the legalization of sports betting in the United States has led to the rising commercial value of sports event data, with many fans relying on real-time data on the matches provided by the betting companies to make their odds and place their bets, making the U.S. sports betting industry only five-year cumulative betting turnover as high as US$220 billion^33,34^. The demand for sports event data has expanded with the introduction of many international sports events and IPs in China. According to a third-party survey, the scale of China's sports competition and performance activities in 2020 will be about 31.84 billion yuan. The scale of sports live-streaming users will be 138 million, with a trend of sustained growth. The continuous expansion of market demand has given rise to many data service enterprises, which has caused the market competition structure in this field to undergo significant changes^32^. Under the multiple competition structure, sports event data service enterprises face not only pressure among peers but also the threat of unfair competitors; at this time, sports event data service enterprises often seek the support and protection of the existing legal framework.

As macro-control tools, government support and regulation will affect the effectiveness of policy implementation and enforcement of data protection^25^. For example, BestData, as the official data service provider of CBA, sued Shanghai Xuanti Co. for using web crawler technology to copy the data of the CBA league. Finally, the court ruled that Shanghai Xuanti Co. damaged the rights and interests of Best Data and the public interest, which constituted unfair competition. However, when the government regulation is unfavorable, or the existing laws and regulations cannot respond effectively to the relevant acts, the interests of the relevant right holders will suffer losses. For example, in the case of NBA v. Motorola, under U.S. law, the court ruled that the information collected in real-time during the event broadcast had entered the public domain and was no longer protected by law^35^. Given the low threshold of sports event piracy, colossal revenue, and the high cost of copyright holders to defend their rights, sports event data service providers (copyright holders) often choose to "turn a blind eye". In this regard, government support and regulation play a crucial role in protecting the rights of sports event data.

- H5: The tournament organizer data management system has a positive effect on enterprise data protection effectiveness.
- H6: Government support has a positive effect on enterprise data protection effectiveness.
- H7: Government regulation has a positive effect on enterprise data protection effectiveness.

**Figure 1:**
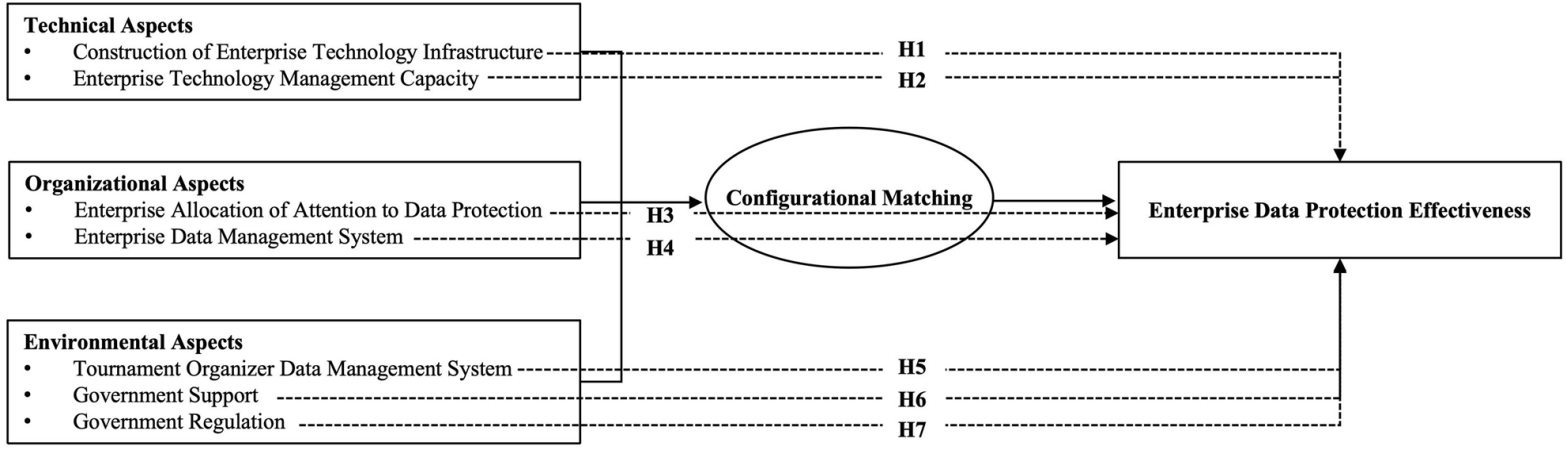
Conceptual framework.

In summary, this study refers to the TOE framework. It combines the practical scenarios of data protection in sports events to deduce seven secondary conditions from the latitude of technical, organizational, and environmental conditions. It constructs a multilevel analysis framework affecting the safety of commercial data rights in sports events (Fig. 1).

## Methodology

### Data collection and measurement

#### Questionnaire design and data collection

Given the limited information on cases of data rights protection in sports events, the study used a questionnaire to collect relevant data. The survey was administered by applicable guidelines and regulations and was reviewed and approved by the School of Economics and Management at Chengdu Sport University. All questionnaire items (26 in total) and corresponding responses (mainly in Likert scales) are listed in the Appendix. The questionnaire was sent electronically via online platforms (including WeChat and Questionnaire Star), and respondents voluntarily clicked on the link to complete the questionnaire. They were informed that by "submitting answers" they were deemed to have given informed consent and that the data they met would be used only for this study, and anonymity was guaranteed. Sample selection followed the case selection principle of the QCA method and was conducted by random sampling. The study first screened a list of all eligible enterprises in China, with the screening condition being that the enterprise business scope was based on the provision of event data services. Stratified sampling was conducted according to the size of the enterprises, and questionnaires were administered. Considering the influence of different sports on the survey results, the study selects sports that apply more data services in China, such as Huaying Sports and Best Data. The questionnaire is filled in by the relevant persons in charge of 15 enterprises and their staff. Since the fsQCA method is more suitable for small and medium-sized samples, and the quality of the questionnaire needs to be strictly demanded, the study adopts a combination of offline and online questionnaires to conduct the research. 150 questionnaires were distributed, and the data were cleaned by checking duplicate IDs, setting up questionnaire answers, etc. At the same time, 138 valid questionnaires were obtained, with an effective recovery rate of 92% after excluding invalid questionnaires such as incomplete questions, most questions with extreme values, and all the questions with the same value (Supplementary Information 1).

#### Variable measurement

This study involves a total of eight variables, and to ensure the validity and reliability of the study, all the measurement variables for measuring the antecedent variables were taken from the existing established scales and fine-tuned accordingly to the circumstances of the content of this study. Among them, the construction of enterprise technology infrastructure (ETI) and enterprise technology management capacity (ETM) refers to the study of Lu and Ramamurthy^36^, which contains eight measurement items. The enterprise allocation of attention to data protection (EAD) and enterprise data management system (EMS) were adapted from the studies of Wang et al.^37^ and Lu and Ramamurthy^36^, which contained a total of 7 measurement questions. The tournament organizer data management system (OMS), government support (GS), and government regulation (GR) were adapted from the study by Wang et al.^37^ and contained a total of 8 items. The measurement items of enterprise data protection effectiveness (EPE) are adapted from the study of Krasnova et al.^38^, which contains 3 items. The measurement items of all scales were set up using the Likert-5 scale, with 1 to 5 representing increasing levels of agreement (1 = strongly disagree, 5 = strongly agree) (Tables 1, 2).

**Table 1:** Research variables.

	Construct	Coding	References
Technical Aspects	Construction of Enterprise Technology Infrastructure	ETI	Lu and Ramamurthy (2011)
	Enterprise Technology Management Capacity	ETM	
Organizational Aspects	Enterprise Allocation of Attention to Data Protection	EAD	Wang et al. (2019)
	Enterprise Data Management System	EMS	Lu and Ramamurthy (2011)
Environmental Aspects	Tournament Organizer Data Management System	OMS	Wang et al. (2019)
	Government Support	GS	
	Government Regulation	GR	
Outcome variable	Enterprise Data Protection Effectiveness	EPE	Krasnova et al. (2010)

**Table 2:** Construct and Indicator Items.

Construction of Enterprise Technology Infrastructure (ETI)	
1. We have strong capabilities in data collection construction (e.g., data collection at the game site or based on game video, etc.)	
2. We have strong capabilities in data integration construction (e.g., checking collected data sources through proprietary applications to validate and ensure accurate data flow)	
3. We have strong capabilities in data center construction (e.g., MySql database as the infrastructure for storing, transferring, recalling, and managing race data)	
4. We have strong capabilities in data distribution construction (e.g., tournament race data, post-tournament ranking updates, daily data updates, end-of-tournament updates, customized distribution, and services, etc.)	
Enterprise Technology Management Capacity (ETM)	
5. We continue to keep up with digital technology innovations	
6. We have the ability and continue to experiment with new digital technologies when necessary	
7. We have a supportive atmosphere for experimenting with new ways of using digital technology	
8. We are constantly looking for new ways to improve the efficiency of using digital technologies	
Enterprise Allocation of Attention to Data Protection (EAD)	
9. We support investment in data protection	
10. We are willing to take the risk of using event data	
11. We may be interested in establishing a data protection system to gain a competitive advantage	
12. We may consider a data protection system as a strategic weapon	
Enterprise Data Management System (EMS)	
13. We develop a clear vision of how digital technologies contribute to business value	
14. We improve the ability of functional areas and general management to understand the value of digital technology investments	
15. We establish an effective and flexible digital transformation planning process and develop a robust digital transformation program	
Tournament Organizer Data Management System (OMS)	
16. The tournament organizer has formulated relevant rules to clarify the content of data authorization	
17. The tournament organizer has formulated relevant rules to clarify the use of data	
18. The tournament organizer requires us to protect data security	
Government Support (GS)	
19. The government has initiated some programs to promote enterprises to carry out data protection	
20. The government has set up some favorable policies for enterprises that carry out data protection	
21. The government will provide financial support to enterprises that plan to carry out data protection	
Government Regulation (GR)	
22. The government makes relevant rules to force us to perform data protection	
23. The government makes relevant rules to penalize enterprises that fail to fulfill their data protection obligations	
Enterprise Data Protection Effectiveness (EPE)	
24. We are confident that we can control all data collected and processed about sporting events	
25. Our data privacy settings allow data right holders full control over the sporting event data they provide	
26. We can control the people who have access to the database to view sporting event data	

After completing the first draft of the questionnaire, the study conducted a sample size of 50 with the survey to conduct the questionnaire reliability test to ensure the validity of the questionnaire. SPSS Statistics 25.0 was used to run the reliability test, and the reliability was determined by Cronbach's $\mathrm{\alpha }$ ($\mathrm{C\alpha }$), composite reliability (${\text{CR}}$), and average variance extracted (AVE) to evaluate. Both CR values are greater than 0.7, and AVE values are more significant than 0.5, indicating that the questionnaire has good internal consistency and is reliable. Usually, the value of the loading coefficient suggests the correlation between the factor and the measurement item; if *p* < 0.01, showing significance, and the value of the loading coefficient is more significant than 0.7, the measurement model meets the basic requirements of structural validity satisfaction. The minimum loading coefficients of this questionnaire are all greater than 0.7, and Bartlett's test results show a significant *p* value of 0, indicating that the questionnaire has good convergent and discriminant validity (Table 3, 4).

**Table 3:** KMO and Bartlett's Test.

KMO	0.813
Approximate Chi-squared value	1047.796
Bartlett’s Test of Sphericity	
* df*	325
* p*	0.000

**Table 4:** Reliability test.

	Construct	$\mathrm{C\alpha }$	CR	AVE	Minimum factor loading
Technical Aspects	Construction of Enterprise Technology Infrastructure (ETI)	0.800	0.801	0.503	0.742
	Enterprise Technology Management Capability (ETM)	0.822	0.822	0.538	0.736
Organizational Aspects	Enterprise Allocation of Attention to Data Protection (EAD)	0.844	0.845	0.578	0.718
	Enterprise Data Management System (EMS)	0.863	0.864	0.680	0.802
Environmental Aspects	Tournament Organizer Data Management System (OMS)	0.821	0.816	0.598	0.704
	Government Support (GS)	0.902	0.903	0.757	0.851
	Government Regulation (GR)	0.853	0.854	0.745	0.849
Outcome Variable	Enterprise Data Protection Effectiveness (EPE)	0.895	0.892	0.736	0.784

#### Correlation and regression analysis

The study used SPSS software to conduct correlation and regression analysis of the condition variables to verify whether a single condition affects the enterprise data protection effectiveness. The study used Pearson's correlation coefficient to indicate the strength of the correlation (Table 5).

**Table 5:** Pearson's correlation coefficient.

			EPE
Technical Aspects	Construction of Enterprise Technology Infrastructure	ETI	0.352**
	Enterprise Technology Management Capability	ETM	0.345**
Organizational Aspects	Enterprise Allocation of Attention to Data Protection	EAD	0.424**
	Enterprise Data Management System	EMS	0.380**
Environmental Aspects	Tournament Organizer Data Management System	OMS	0.382**
	Government Support	GS	0.356**
	Government Regulation	GR	0.314**

*p < 0.05; **p < 0.01.

The results showed that all conditions and EPE showed significance at the 0.01 level, thus indicating a significant positive correlation between the conditions and EPE. Regression analysis was further applied to verify the influence relationship between the variables (Table 6). Table 6 shows that none of the conditions will directly influence EPE, and none of the above hypothesis are valid. Through F-test (F = 6.687, *p* = 0.000 < 0.05), the study found that at least one of the conditions would impact EPE. In protecting data rights in sports events, the conditions do not function independently but rather achieve adequate data protection through the linkage of reinforcement or counteracting.

**Table 6:** Regression analysis results.

	Unstandardized Regression Coefficient		Standardized Regression Coefficient	*t*	*p*	Covariance Diagnostics	
	*B*	S.E	*Beta*			VIF	TOL
C	0.839	0.37	–	2.268	0.025*	–	–
ETI	0.085	0.187	0.082	0.457	0.649	5.663	0.177
ETM	0.087	0.177	0.087	0.494	0.622	5.534	0.181
EAD	0.265	0.16	0.238	1.653	0.101	3.666	0.273
EMS	0.054	0.148	0.051	0.366	0.715	3.454	0.29
OMS	0.218	0.159	0.206	1.369	0.173	3.988	0.251
GS	0.124	0.16	0.12	0.773	0.441	4.262	0.235
GR	-0.116	0.153	− 0.115	− 0.758	0.45	4.041	0.247
*R* ^2^	0.265						
Adjusted R ^2^	0.225						
*F*	*F* (7,130) = 6.687, *p* = 0.000						
D-W value	1.696						

DV: EPE

*p < 0.05; **p < 0.01.

### Data analysis and empirical results

#### Variable calibration

The study obtained the data related to each variable based on a questionnaire, each variable contains 2–4 measurement question items. The study used the average score of each variable as the final score of the variable and constructed the calibration anchors by referring to the studies of Fiss^39^ and Greckhamer^40^, respectively, and selected the upper quartile of the sample's descriptive statistics, the median and the lower quartile as the total affiliation threshold, intersection point, and full non-affiliation threshold. The anchor points for each variable were calculated using the Percentile function (Table 7), and the integrated data were calibrated using the Calibrate function in fsQCA4.1.

**Table 7:** Variable calibration anchor point.

Construct	Anchor point		
	Full affiliation value	Junction	Totally unaffiliated values
ETI	4.001	3.251	2.501
ETM	4.251	3.251	2.501
EAD	4.001	3.251	2.501
EMS	4.001	3.334	2.668
OMS	4.334	3.668	2.668
GS	4.334	3.668	2.668
GR	4.001	3.501	2.501
EPE	4.001	3.334	2.334

Necessity conditions are the conditions that must exist to cause an outcome to occur and are the basis for performing a fuzzy set truth table procedure analysis^41^. Since truth table analysis is in essence sufficiency analysis, detecting the presence of a single necessity condition is a prerequisite for carrying out the next step in the analysis. Conditions with consistency greater than 0.9 are generally regarded as necessity conditions, indicating that the antecedent condition is necessary for the protection of sports event data rights. Whereas conditions less than 0.9 do not constitute a necessity condition^42^, indicating that the antecedent condition has weak explanatory power for the protection of data rights in sports events^43^. According to the results of the analysis, none of the antecedent conditions can constitute a necessary condition for the protection of sports event data rights in individual cases, indicating that the protection of sports event data rights is affected by the intertwining of multiple factors (Table 8).

**Table 8:** Necessity analysis of antecedent variables.

Construct	EPE		~ EPE	
	Consistency	Coverage	Consistency	Coverage
ETI	0.633947	0.614383	0.478861	0.471404
~ ETI	0.454572	0.461996	0.608283	0.627969
ETM	0.620070	0.619980	0.453408	0.460494
~ ETM	0.460415	0.453330	0.625827	0.625917
EAD	0.665352	0.646742	0.476848	0.470822
~ EAD	0.455595	0.461596	0.642220	0.660944
EMS	0.618463	0.622281	0.452258	0.462228
~ EMS	0.465528	0.455546	0.630428	0.626644
OMS	0.630733	0.670705	0.394162	0.425753
~ OMS	0.459977	0.427737	0.695139	0.656615
GS	0.630149	0.664920	0.416595	0.446517
~ GS	0.475460	0.445159	0.687374	0.653720
GR	0.613643	0.620899	0.447944	0.460390
~ GR	0.466696	0.454223	0.631147	0.623969

Based on the analysis of individually necessary conditions, the study used fsQCA4.1 to analyze the fuzzy set truth table of the integrated data and chose a case frequency threshold of 1 and a consistency threshold of more than 0.8^39^. To avoid the problem of a "simultaneous subset relationship", the PRI value was kept above 0.7^41^. Through the standard analysis, complex solutions (without logical residuals), parsimonious solutions (with logical residuals but without evaluating their reasonableness), and intermediate solutions (with logical residuals but only those that are reasonably justified) are obtained. Typically, the conditions that appear in the parsimonious solution are referred to as core conditions. In contrast, the remaining conditions that appear in the intermediate solution but not in the parsimonious solution are referred to as peripheral conditions, i.e., those that have a weak causal relationship with the outcome^41^. The result is the seven configurational conditions for realizing high and low enterprise data protection effectiveness (Table 9).

**Table 9:**
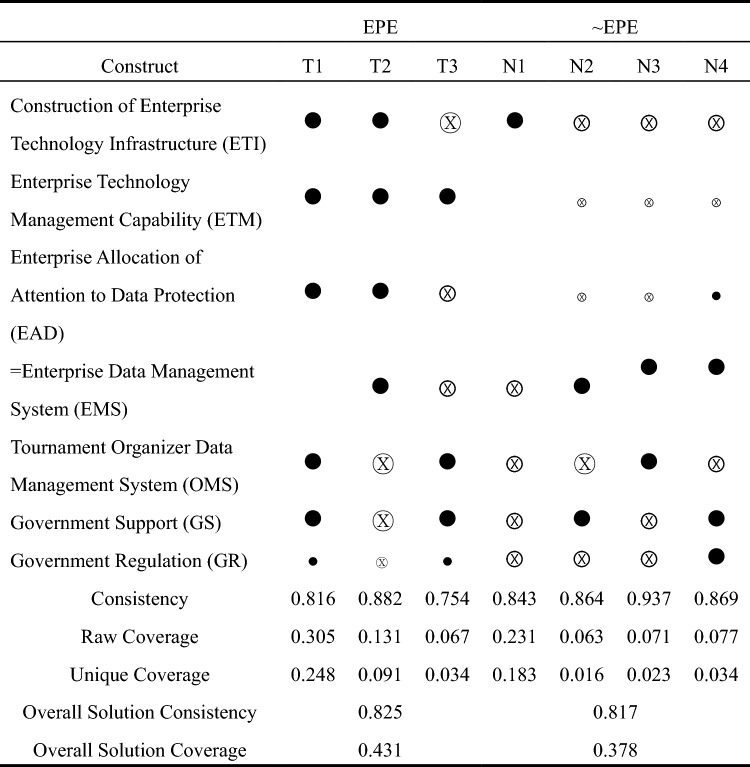
Configuration of influencing factors of data rights protection for sports events.

Black circle (●) indicate the presence of a condition, and circles with “x” (Ⓧ) indicate its absence. Large circle; core condition. Small circle; peripheral condition. Blank space; “don’t care” condition^44^.

As can be seen from Table 9, the seven configurational conditions sports event data rights protection have an overall solution consistency of 0.825, an overall solution coverage of 43.1%, and the consistency values of the single solutions are 0.816, 0.882, 0.754, 0.843, 0.864, 0.937, 0.869, which are all over the minimum threshold value of 0.75^42^, which indicates that the seven configurational conditions are the sports event data rights protection sufficiency condition, its reliability is good, and its explanation of the outcome variables is strong.

To better identify the differences between the different conditions, the study categorizes the conditions that achieve high enterprise data protection effectiveness as All-Factor-Driven (T1), Technology-Organization-Supporting (T2), and Technology-Environment-Driven (T3). The conditions for achieving low enterprise data protection effectiveness are categorized as Organizational-Environmental-Deficient (N1) and Technology-Deficient (N2, N3, N4), which are further explained below ("*" indicates that each antecedent is present at the same time; "~" indicates that the antecedent is not present)^25^.

**Configuration T1:** ETI*ETM*EAD*OMS*GS*GR, where EMS may be present or absent. This conditional configuration shows that when all the antecedent factors exist, the data protection effectiveness of sports events can be strengthened. However, the existence of all antecedent factors implies that the data service enterprise not only has complete construction of enterprise technical facilities and enterprise technical management ability but also has enough attention allocated to data protection and a perfect enterprise data management system; at the same time, there is also a good external environment, including the management system of event organizers, the support of the government departments and regulatory efforts. Therefore, this configuration path is called "All-Factor-Driven", but in actual practice, the possibility of the simultaneous existence of all the antecedent factors is low.

**Configuration T2:** ETI*ETM*EAD*EMS* ~ OMS* ~ GS* ~ GR shows that if the data service enterprise has reasonable technical and organizational conditions, regardless of the external environment (Tournament organizer data management system, government support, government regulation), it can realize adequate protection of the event data rights. In this regard, the study calls this configuration path "Technology-Organization-Supporting". However, it should be noted that this condition configurational has higher requirements for the enterprise's strength, i.e., the enterprise needs to have good construction of enterprise technology infrastructure and enterprise technology management capability and a perfect organization management system.

**Configuration T3: **~ ETI*ETM* ~ EAD* ~ EMS*OMS*GS*GR, indicating that in the absence of institutional conditions for data management in data service enterprises, when data service enterprises have good enterprise technology management capability, regardless of whether or not they have good construction of enterprise technology infrastructure, as long as the data service enterprises can respond quickly to external changes and serve the strategic decisions of the enterprises, coupled with solid support and strict supervision by the government, the data service enterprises are also able to strengthen the effectiveness of tournament data protection to a certain extent. Suppose the data service enterprises can respond quickly to external changes and serve the strategic decision-making of the enterprises, coupled with the government's strong support and strict supervision. In that case, the data service enterprises can also strengthen the effectiveness of event data protection to a certain extent. In this regard, the study calls this configuration path "Technology-Environment-Driven", which emphasizes the importance of technological and external environmental conditions to the data protection effectiveness of enterprises.

**Configuration N1:** ETI* ~ EMS* ~ OMS* ~ GS* ~ GR, this conditional configuration shows that only having good corporate technical facilities without the support of organizational and external environmental conditions will result in low corporate data protection effectiveness. This configuration path is called "Organizational-Environmental-Deficient", which emphasizes the importance of organizational conditions and the external environment. To protect enterprise data effectively, the importance of these two aspects should be addressed.

**Configuration N2, N3, N4:** ~ ETI* ~ ETM* ~ EAD*EMS* ~ OMS*GS* ~ GR; ~ ETI* ~ ETM* ~ EAD*EMS*OMS* ~ GS* ~ GR; ~ ETI* ~ ETM*EAD*EMS* ~ OMS*GS*GR. The above three configurations all reflect the same characteristic, i.e., the lack of technology conditions. It shows that even if enterprises and event organizers have a perfect organization and management system, and government support and regulation are strong if the technical conditions are missing, it will produce low effectiveness of enterprise data protection. In this regard, the study refers to the above three conditions as "Technology-Deficient", suggesting that technological conditions are key to improving enterprise data protection effectiveness.

### Robustness testing

To ensure the robustness of the findings, two robust-type tests were conducted in this study. For the first test, the consistency threshold was adjusted from 0.8 to 0.85. The results were generally consistent, with the overall consistency of achieving high enterprise data protection effectiveness increasing from 0.825 to 0.831, and the overall consistency of achieving low enterprise data protection effectiveness rising from 0.817 to 0.848. For the second test, the study increased the PRI consistency from 0.7 to 0.75, with all the resulting conditional configuration remaining the same. This show that the results of the study were stable.

## Conclusions and limitations

### Conclusions

Factors affecting the protection of sports event data rights are not independent and single, but a variety of factors interact with each other, and existing research is limited by the methodology, and the causal relationship between each influencing factor and the protection of sports event data rights is unclear. Therefore, this chapter analyzes the combination of antecedent factors of sports event data rights protection based on configurational theory and using the fsQCA method. The study obtains three configuration paths of "All-Factor-Driven", "Technology-Organization-Supporting" and "Technology-Environment-Driven" to achieve high enterprise data protection effectiveness. The two configuration paths to achieve low enterprise data protection effectiveness are "Organizational-Environmental-Deficient" and "Technology-Deficient". Among them, the "All-Factor-Driven" is to influence the high enterprise data protection effectiveness through the factors of technology level, organization level, and environment level together; the "Technology-Organization-Supporting" is to control the high enterprise data protection effectiveness through the factors of technology level and organization level; The "Technology-Environment-Driven" approach emphasizes the influence of technological and environmental factors on high enterprise data protection effectiveness. Meanwhile, "Organizational-Environmental-Deficient" and "Technology-Deficient" indicate low corporate data protection effectiveness when the technological and environmental dimensions are absent, or the technical dimension is missing.

### Main contributions

The main contributions of this study are in both theory and practice.

At the theoretical level, unlike the focus of most previous studies, this study takes as its entry point the exploration of the protection of data rights in sports events, an area that is currently the subject of much-needed in-depth discussion. Second, the study introduces configurational theory and fsQCA methodology, which provides a new research framework and research path for this field. Unlike traditional qualitative research methods, configurational theory provides an effective end to the problem of multi-factor concurrent causal complexity, which provides a basis for the study to seek the key combinations of factors affecting the protection of data rights in sports events.

On the practical level, the practical significance and social impact of this study is mainly reflected in three aspects. First, the cooperation and mutual trust of all parties in the protection of sports event data rights can promote the cultivation of the sports event data factor market and further promote the commercial value of sports event data. Second, the findings of this study provide direction and support for improving the institutional management system of sports event organizers and data service enterprises and provide institutional strength for better optimizing the system of sports event data rights protection. Third, exploring and promoting the characteristic physical mechanism is a practical initiative supported by the conclusions of this study.

### Limitations and future research

Firstly, the division of stakeholders around the protection of interests in sports event data can be further refined and deepened. According to Freeman (1983), stakeholders are divided into three aspects: ownership stakeholders, economically dependent stakeholders, and social stakeholders. Some scholars have adopted different divisions, such as Charkham (1992), who divided stakeholders into contractual and public stakeholders based on the existence of a contractual relationship^45^. Wheeler (1998), on the other hand, divided relationship stakeholders into primary social stakeholders, secondary social stakeholders, and primary non-social stakeholders from the perspectives of both social and relatedness. stakeholders, primary non-social stakeholders, and secondary non-social stakeholders^46^. Therefore, future research can start from different perspectives to discuss the detailed division of stakeholders in the protection of sports event data rights.

Secondly, configurational analysis is mainly used in the analysis of the realization path of the protection of sports event data rights, and the research data is obtained through the questionnaire survey method to serve as the basis for the subsequent research. However, the information obtained by the questionnaire survey method is still limited, so the subsequent research can carry out more in-depth qualitative analysis through the interview method and case study method. In addition, future research can try to introduce new methods to conduct quantitative analysis based on configurational analysis.

Finally, further discussion can be carried out on the institutional restructuring of the protection of data rights and interests in sports events, introducing the theory of institutional logic and exploring the failure of the protection of data rights and interests in sports events from the perspective of multiple logical conflicts^47^.

### Supplementary Information

Supplementary Information.

## Data Availability

All relevant data are within the paper and its Supporting Information files.
