# Supplementary material for: Factors affecting the protection of data rights in sports events: a configurational analysis
Source: Sci Rep. 2024 Mar 4;14:5353. doi: 10.1038/s41598-024-56074-6 (PMC10912312; doi:10.1038/s41598-024-56074-6)
Supplement: Supplementary file 1 — Supplementary Information. [file 41598_2024_56074_MOESM1_ESM.docx]

**Supplementary material**

**Questionnaire. All data in units.**

| **1 We have strong capabilities in data collection construction (e.g., data collection at the game site or based on game video, etc.).** | **END** |
| --- | --- |
| Strongly Disagree | 5 |
| Disagree | 35 |
| General | 39 |
| Agree | 35 |
| Strongly Agree | 24 |
| **2 We have strong capabilities in data integration construction (e.g., checking collected data sources through proprietary applications to validate and ensure accurate data flow).** | **END** |
| Strongly Disagree | 7 |
| Disagree | 34 |
| General | 34 |
| Agree | 34 |
| Strongly Agree | 29 |
| **3 We have strong capabilities in data center construction (e.g., MySql database as the infrastructure for storing, transferring, recalling, and managing race data).** | **END** |
| Strongly Disagree | 5 |
| Disagree | 37 |
| General | 28 |
| Agree | 37 |
| Strongly Agree | 31 |
| **4 We have strong capabilities in data distribution construction (e.g., tournament race data, post-tournament ranking updates, daily daily data updates, end-of-tournament updates, customized distribution and services, etc.)** | **END** |
| Strongly Disagree | 4 |
| Disagree | 44 |
| General | 21 |
| Agree | 37 |
| Strongly Agree | 32 |
| **5 We continue to keep up with digital technology innovations.** | **END** |
| Strongly Disagree | 5 |
| Disagree | 36 |
| General | 26 |
| Agree | 35 |
| Strongly Agree | 36 |
| **6 We have the ability and continue to experiment with new digital technologies when necessary.** | **END** |
| Strongly Disagree | 6 |
| Disagree | 42 |
| General | 20 |
| Agree | 38 |
| Strongly Agree | 32 |
| **7 We have a supportive atmosphere for experimenting with new ways of using digital technology.** | **END** |
| Strongly Disagree | 4 |
| Disagree | 38 |
| General | 35 |
| Agree | 35 |
| Strongly Agree | 26 |
| **8 We are constantly looking for new ways to improve the efficiency of using digital technologies.** | **END** |
| Strongly Disagree | 7 |
| Disagree | 39 |
| General | 29 |
| Agree | 33 |
| Strongly Agree | 30 |
| **9 We support investment in data protection.** | **END** |
| Strongly Disagree | 1 |
| Disagree | 43 |
| General | 32 |
| Agree | 38 |
| Strongly Agree | 24 |
| **10 We are willing to take the risk of using event data.** | **END** |
| Strongly Disagree | 5 |
| Disagree | 37 |
| General | 33 |
| Agree | 38 |
| Strongly Agree | 25 |
| **11 We may be interested in establishing a data protection system to gain a competitive advantage.** | **END** |
| Strongly Disagree | 8 |
| Disagree | 26 |
| General | 40 |
| Agree | 40 |
| Strongly Agree | 24 |
| **12 We may consider a data protection system as a strategic weapon.** | **END** |
| Strongly Disagree | 3 |
| Disagree | 39 |
| General | 30 |
| Agree | 41 |
| Strongly Agree | 25 |
| **13 We develop a clear vision of how digital technologies contribute to business value.** | **END** |
| Strongly Disagree | 5 |
| Disagree | 34 |
| General | 31 |
| Agree | 37 |
| Strongly Agree | 31 |
| **14 We improve the ability of functional areas and general management to understand the value of digital technology investments.** | **END** |
| Strongly Disagree | 5 |
| Disagree | 39 |
| General | 33 |
| Agree | 38 |
| Strongly Agree | 23 |
| **15 We establish an effective and flexible digital transformation planning process and develop a robust digital transformation program.** | **END** |
| Strongly Disagree | 6 |
| Disagree | 31 |
| General | 29 |
| Agree | 46 |
| Strongly Agree | 26 |
| **16 The tournament organizer has formulated relevant rules to clarify the content of data authorization.** | **END** |
| Strongly Disagree | 2 |
| Disagree | 37 |
| General | 29 |
| Agree | 36 |
| Strongly Agree | 34 |
| **17 The tournament organizer has formulated relevant rules to clarify the use of data.** | **END** |
| Strongly Disagree | 4 |
| Disagree | 31 |
| General | 33 |
| Agree | 44 |
| Strongly Agree | 26 |
| **18 The tournament organizer requires us to protect data security.** | **END** |
| Strongly Disagree | 6 |
| Disagree | 26 |
| General | 34 |
| Agree | 42 |
| Strongly Agree | 30 |
| **19 The government has initiated some programs to promote enterprises to carry out data protection.** | **END** |
| Strongly Disagree | 6 |
| Disagree | 28 |
| General | 33 |
| Agree | 45 |
| Strongly Agree | 26 |
| **20 The government has set up some favorable policies for enterprises that carry out data protection.** | **END** |
| Strongly Disagree | 4 |
| Disagree | 38 |
| General | 25 |
| Agree | 33 |
| Strongly Agree | 38 |
| **21 The government will provide financial support to enterprises that plan to carry out data protection.** | **END** |
| Strongly Disagree | 3 |
| Disagree | 33 |
| General | 33 |
| Agree | 36 |
| Strongly Agree | 33 |
| **22 The government makes relevant rules to force us to perform data protection.** | **END** |
| Strongly Disagree | 6 |
| Disagree | 27 |
| General | 36 |
| Agree | 36 |
| Strongly Agree | 33 |
| **23 The government makes relevant rules to penalize enterprises that fail to fulfill their data protection obligations.** | **END** |
| Strongly Disagree | 6 |
| Disagree | 33 |
| General | 39 |
| Agree | 35 |
| Strongly Agree | 25 |
| **24 We are confident that we can control all data collected and processed about sporting events.** | **END** |
| Strongly Disagree | 5 |
| Disagree | 38 |
| General | 29 |
| Agree | 38 |
| Strongly Agree | 28 |
| **25 Our data privacy settings allow data right holders full control over the sporting event data they provide.** | **END** |
| Strongly Disagree | 6 |
| Disagree | 43 |
| General | 29 |
| Agree | 33 |
| Strongly Agree | 27 |
| **26 We can control the people who have access to the database to view sporting event data.** | **END** |
| Strongly Disagree | 6 |
| Disagree | 40 |
| General | 33 |
| Agree | 36 |
| Strongly Agree | 23 |
